# Hydrotropic Solubilization of Lipophilic Drugs for Oral Delivery: The Effects of Urea and Nicotinamide on Carbamazepine Solubility–Permeability Interplay

**DOI:** 10.3389/fphar.2016.00379

**Published:** 2016-10-25

**Authors:** Avital Beig, David Lindley, Jonathan M. Miller, Riad Agbaria, Arik Dahan

**Affiliations:** ^1^Department of Clinical Pharmacology, School of Pharmacy, Faculty of Health Sciences, Ben-Gurion University of the NegevBeer-Sheva, Israel; ^2^AbbVie Incorporation, North ChicagoIL, USA

**Keywords:** drug absorption, hydrotropic solubilization, intestinal permeability, oral drug delivery, solubility

## Abstract

Hydrotropy refers to increasing the water solubility of otherwise poorly soluble compound by the presence of small organic molecules. While it can certainly increase the apparent solubility of a lipophilic drug, the effect of hydrotropy on the drugs’ permeation through the intestinal membrane has not been studied. The purpose of this work was to investigate the solubility–permeability interplay when using hydrotropic drug solubilization. The concentration-dependent effects of the commonly used hydrotropes urea and nicotinamide, on the solubility and the permeability of the lipophilic antiepileptic drug carbamazepine were studied. Then, the solubility–permeability interplay was mathematically modeled, and was compared to the experimental data. Both hydrotropes allowed significant concentration-dependent carbamazepine solubility increase (up to ∼30-fold). A concomitant permeability decrease was evident both *in vitro* and *in vivo* (∼17-fold for nicotinamide and ∼9-fold for urea), revealing a solubility–permeability tradeoff when using hydrotropic drug solubilization. A relatively simplified simulation approach based on proportional opposite correlation between the solubility increase and the permeability decrease at a given hydrotrope concentration allowed excellent prediction of the overall solubility–permeability tradeoff. In conclusion, when using hydrotropic drug solubilization it is prudent to not focus solely on solubility, but to account for the permeability as well; achieving optimal solubility–permeability balance may promote the overall goal of the formulation to maximize oral drug exposure.

## Introduction

In recent years, modern drug discovery efforts have been producing more and more lipophilic drug candidates, and according to some estimates more than 50% of new drug entities exhibit poor water solubility (Lipinski et al., 2001; Dahan et al., 2013b; Pham-The et al., 2013; Wolk et al., 2014). According to the biopharmaceutics classification system (BCS; Amidon et al., 1995), the oral absorption of these drugs may be limited by their solubility/dissolution in the aqueous gastrointestinal (GI) milieu (Lobenberg and Amidon, 2000; Martinez and Amidon, 2002; Dahan et al., 2009). The use of different solubility-enabling formulations is a very common practice in tackling solubility limitations, however, in recent years, it was reported that care should be taken when using these formulations; in some cases it was evident that the increased solubility afforded by the formulations in accompanied by a parallel decreased intestinal permeability (Dahan and Miller, 2012; Beig et al., 2015b). Since solubility and permeability together were identified as the key factors dominating oral drug absorption (Lennernas, 1998; Lennernäs and Abrahamsson, 2005; Dahan et al., 2010a, 2012), this tradeoff may jeopardize the ability of a given formulation to improve the overall absorption. For instance, the use of cyclodextrin-based formulations was associated with such solubility–permeability tradeoff (Dahan et al., 2010b; Beig et al., 2013a, 2015a). Likewise, surfactants (Miller et al., 2011; Hens et al., 2015) and cosolvent-based delivery systems resulted in permeability decrease concomitantly to the solubility increase (Beig et al., 2012; Miller et al., 2012b). On the other hand, no such tradeoff was observed with amorphous solid dispersions (ASD) irrespective of the supersaturation level attained (Miller et al., 2012a; Dahan et al., 2013a, 2016). This fundamental difference between different solubilization methods and ASD formulations represent a significant advantage of the latter, as ASD may increase the drug flux across the intestinal barrier (which is the product of solubility times permeability) more easily than the above mentioned solubilization techniques.

Hydrotropy is an emerging powerful drug solubilization strategy, which has been shown to significantly improve the solubility of many drugs (Booth et al., 2012). Hydrotropy refers to the process by which a large amount of solute (the hydrotrope) enhances the solubility of another compound (the drug), by a mechanism that is not yet fully understood (Shimizu et al., 2013). Hydrotropic agents are typically characterized by an amphiphilic molecular structure, however, they are distinguished from surfactants because their hydrophobicity is not sufficient to produce well organized self-associated aggregates such as micelles (Hodgdon and Kaler, 2007; Eastoe et al., 2011; Booth et al., 2015). In fact, self-aggregation of the hydrotrope molecules hampers the hydrotropy capacity (Booth et al., 2012; Shimizu et al., 2013). Hydrotropic drug solubilization is also mechanistically different from cosolvency. For instance, while cosolvents enhance drug solubility by minimizing the polarity gap between the solvent and the drug, it was reported that hydrotropes preferentially concentrate nearby the drug molecules (da Silva et al., 1999). It is likely that the combination of a number of mechanisms responsible for hydrotropic drug solubilization, including the depression of water activity and drug-hydrotrope interactions (Shimizu et al., 2013). Overall, it emerges that hydrotropy is a distinctive standalone drug solubilization technique, which may dramatically increase the apparent solubility of drugs (Agrawal et al., 2004; Kim et al., 2010, 2011).

Hydrotropes are frequently anionic aromatic (e.g., salicylate, benzoate) or non-aromatic compounds (e.g., citrate), but can also be neutral (e.g., urea; Herbig and Evers, 2013). Urea is widely used as a hydrotropic agent, as it was shown to enhance the aqueous solubility of many lipophilic drugs including diclofenac (250-fold), hydrochlorothiazide (74-fold), and many others (Coffman and Kildsig, 1996a,b). Urea constitutes a near-ideal mixture with water; in contrast to several other hydrotropes, urea exhibits weak self-association in water, and since hydrotrope self-association reduces the solubilization capacity, urea exhibits very minor loss of solubilization efficiency (Booth et al., 2012, 2015). Nicotinamide, the product of nicotinic acid (niacin) *in vivo* conversion, is another effective and commonly used hydrotrope, which has been demonstrated to solubilize a wide variety of lipophilic drugs (Sanghvi et al., 2007; Cui et al., 2010; Booth et al., 2015).

The primary purpose of this work was to investigate the solubility–permeability interplay when using hydrotropic drug solubilization; revealing whether hydrotropy generates solubility–permeability tradeoff (similarly to cyclodextrins, surfactants and cosolvents), or it affords solubility increase without hampering the permeability (similarly to ASD formulations), is critical to the overall use of this technique. We have selected the two commonly used hydrotropes urea and nicotinamide, and studied their concentration-dependent effects on the solubility, the *in vitro* and *in vivo* permeability, and the solubility–permeability interplay, of the lipophilic antiepileptic drug carbamazepine. Then, we made an effort to allow *a priori* computational prediction of the solubility–permeability interplay when using hydrotropic drug solubilization, to facilitate the development of an optimized formulation. Overall, this work aimed to reveal significant mechanistic insights regarding the use of hydrotropic solubilization in oral delivery of lipophilic drugs.

## Materials and Methods

### Materials

Urea, nicotinamide, carbamazepine, and MES buffer were purchased from Sigma Chemical Co. (St. Louis, MO, USA). Potassium chloride and sodium chloride were obtained from Fisher Scientific Inc. (Pittsburgh, PA, USA). Acetonitrile, methanol and water (Merck KGaA, Darmstadt, Germany) were UPLC grade. All other chemicals were of analytical reagent grade.

### Solubility

Carbamazepine solubility was measured at increasing concentrations (0–40% w/v) of hydrotrope (urea vs. nicotinamide) in 10 mM MES buffer, pH 6.5, at room temperature (25°C) and at 37°C as described previously (Fairstein et al., 2013; Zur et al., 2014a). Briefly, excess of drug powder was incubated with different urea/nicotinamide solutions (0–40% w/v) for 24–48 h, followed by centrifugation, supernatant withdrawal, filtration, and UPLC analysis for drug content.

### *In vitro* Parallel Artificial Membrane Permeability Assay (PAMPA) Permeability

Parallel artificial membrane permeability assay (PAMPA) was carried out using a method previously reported (Sun et al., 2009; Zur et al., 2014b). In brief, carbamazepine solutions were prepared with different levels (0–40% w/v) of hydrotrope (urea vs. nicotinamide) in MES buffer pH 6.5. Carbamazepine concentrations in the different urea levels were calculated to achieve 75% saturation in all experimental groups. PAMPA experiments were carried out in 96-well MultiScreen-Permeability filter plates with 0.3 cm^2^ polycarbonate filter support (0.45 mm). The filter supports in each well were first filled with 15 μL of a 5% solution (v/v) of hexadecane in hexane. After evaporation of the hexane (60 min), the carbamazepine-hydrotrope solutions were placed in each donor well, and the receiver wells were filled with blank MES buffer solution. The donor plate was then set upon the receiver plate, and was incubated with shaking at room temperature (25°C). Samples from the receiver wells were collected every 30 min over 2.5 h and were assayed for drug content by UPLC.

The *in vitro* permeability (P_app_; cm/sec) of carbamazepine was calculated from the steady-state drug accumulation in the receiver well (dQ/dt) according to the following equation:

$$P_{\text{app}}=\frac{dQ/dt}{AC_{0}}$$

where *A* is the membrane surface area available for permeation, and *C*_0_ is carbamazepine’s initial concentration in the donor well.

### *In vivo* Permeability Studies in Rats

All animal studies protocols were approved by the Animal Use and Care Committee of Ben-Gurion University of the Negev (Protocol IL-08-01-2015). Animals were housed and handled according to Ben-Gurion University of the Negev Unit for Laboratory Animal Medicine Guidelines. Male 300–330 g Wistar rats (Harlan, Israel) were used for these studies.

The single-pass intestinal perfusion studies were carried out as previously described (Varghese Gupta et al., 2011; Gupta et al., 2013; Lozoya-Agullo et al., 2015). Briefly, carbamazepine solutions were prepared with hydrotrope (0, 10, 20, and 30%, urea vs. nicotinamide) in 10 mM MES buffer (pH 6.5). Drug concentrations in the different urea/nicotinamide levels were calculated according to the solubility data at 37°C to achieve 75% saturation in all experimental groups. A 10 cm segment of the jejunum was cannulated at both ends, and the carbamazepine-hydrotrope solution was perfused through this intestinal segment. Comparison of the inlet vs. the outlet drug concentrations was used to calculate the permeability (P_eff_), according to the following equation (Zur et al., 2015):

$$P_{\text{eff}}=\frac{-Q\ln\left(C_{\text{out}}^{\prime }/C_{\text{in}}^{\prime }\right)}{2\pi RL}$$

where *Q* is the perfusion flow rate (0.2 mL/min), $C_{\mathrm{out}}^{\prime }$/$C_{\mathrm{in}}^{\prime }$ is the ratio of outlet vs. inlet carbamazepine concentrations adjusted to water flux, *R* is the intestinal radius (set to 0.2 cm), and *L* is the length of the perfused jejunal segment, accurately measured at the end of the experiment. Stability of the drug in the intestinal lumen was evaluated to eliminate the option of drug disappearance that is not attributable to absorption.

### Ultra-Performance Liquid Chromatography (UPLC)

Ultra-performance liquid chromatography (UPLC) experiments were performed on a Waters (Milford, MA, USA) Acquity UPLC H-Class system equipped with photodiode array (PDA) detector and Empower software, as previously described in detail (Beig and Dahan, 2014). The mobile phase consisted of 30:70 going to 70:30 (v/v) 0.1% TFA in water: 0.1% TFA in acetonitrile over 8 min, that was pumped at a flow rate of 0.5 mL/min. Injection volumes for all UPLC analyses ranged from 5 to 50 μL, and the detection wavelength was 285 nm.

### Statistical Analysis

Solubility and *in vivo* permeability studies were *n* = 5 and *in vitro* PAMPA permeability experiments were *n* = 6. Data are expressed as mean ± standard deviation (SD). To determine statistically significant differences among the experimental groups, the non-parametric Kruskal–Wallis test was used for multiple comparisons, and the non-parametric Mann–Whitney *U* test for two-group comparison where appropriate. A *p*-value lower than 0.05 was considered significant.

## Results

### Carbamazepine-Hydrotrope Solubility Studies

**FIGURE 1:**
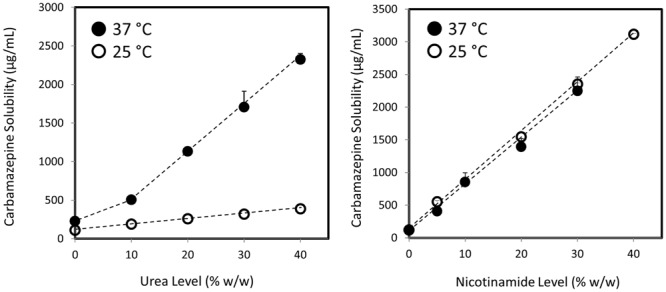
**Apparent solubility (μg/mL) of carbamazepine as a function of increasing urea (left panel) or nicotinamide (right panel) levels at 37°C (∙) and at 25°C (room temperature; o).** Data are presented as mean ± SD; *n* = 5.

Carbamazepine solubility at room temperature (25°C) and at 37°C as a function of hydrotrope concentration is presented in **Figure 1**. Three main findings can be readily seen in **Figure 1**: (1) hydrotropic solubilization of carbamazepine, using either urea or nicotinamide, is a powerful solubilization method that can dramatically increase the drugs’ apparent solubility; (2) while carbamazepine’s solubility in the absence of any excipient is temperature-dependent (the drug’s solubility is more than doubled at 37°C), the two hydrotropes react very differently to temperature variation: it can be seen that the ability of urea to increase carbamazepine solubility is temperature-dependent, while nicotinamide is not; and (3) closer analysis of the effect of urea reveals that while at room temperature the increased solubility appears to be linear as urea level increases, at 37°C carbamazepine shows biphasic solubility trend, with higher slope at hydrotrope (urea) levels above 10% w/v. Analogous findings were recently reported for the hydrotropic solubilization of the lipophilic drugs griseofulvin and estrone (Sanghvi et al., 2007). The effect of nicotinamide appears to be linear at all hydrotrope concentrations studied.

### The Effect of Hydrotrope on Carbamazepine *In vitro* Permeability

**FIGURE 2:**
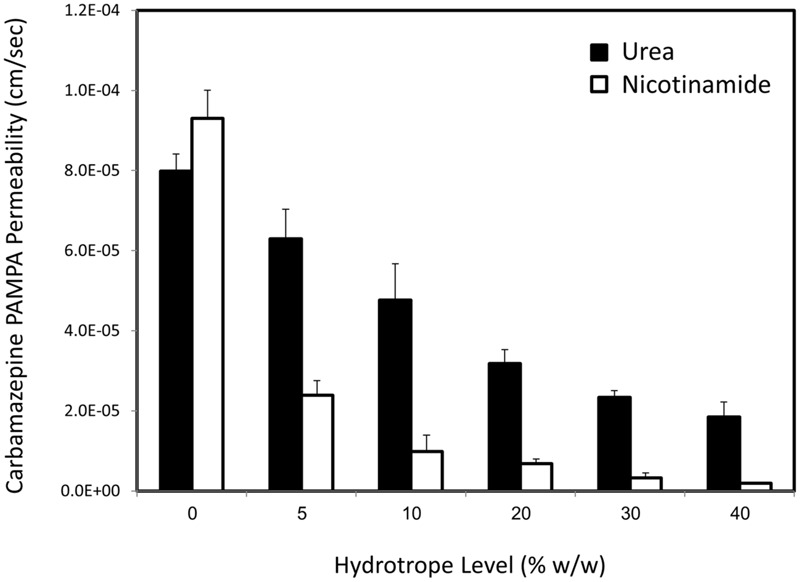
**Experimental apparent *in vitro* PAMPA permeability (cm/sec) of carbamazepine as a function of increasing levels of urea (black bars) or nicotinamide (white bars).** Data are presented as mean ± SD; *n* = 6.

Carbamazepine *in vitro* permeability as a function of increasing levels of urea (black bars) or nicotinamide (white bars) is presented in **Figure 2**. It can be seen that with both hydrotropes the drug’s apparent permeability decreased significantly with increasing hydrotrope levels (and increased apparent solubility) in a concentration- dependent manner. This result indicates that a tradeoff between solubility increase and permeability decrease exists when using hydrotropic drug solubilization, which means that every solubility gain is accompanied by a concomitant permeability loss.

### The Effect of Hydrotrope on Carbamazepine *In vivo* Permeability

**FIGURE 3:**
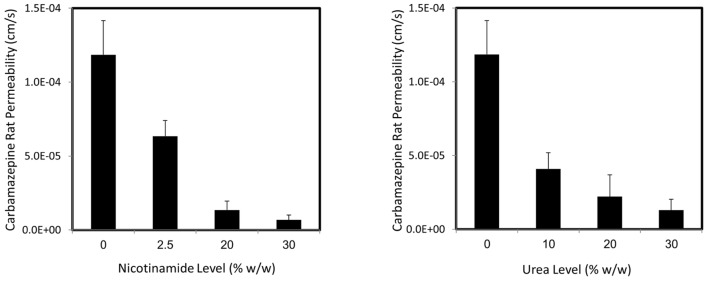
**Carbamazepine *in vivo* permeability as a function of increasing nicotinamide (left panel) or urea levels (right panel) in the single-pass intestinal rat perfusion model.** Data are presented as mean ± SD; *n* = 5.

The rat intestinal permeability of carbamazepine from drug solutions containing increasing levels of nicotinamide (left panel) and urea (right panel) is presented in **Figure 3**. Similarly to the *in vitro* permeability results, significantly decreased effective *in vivo* carbamazepine permeability was found with increasing hydrotrope levels (and increased apparent solubility). Once more, it was revealed that the *in vivo* solubility–permeability interplay when using hydrotropic drug solubilization has the nature of a tradeoff, confirming that the increased apparent solubility via hydrotropy comes with a price tag of simultaneous decreased intestinal permeability.

### Solubility–Permeability Interplay When Using Hydrotropy

**FIGURE 4:**
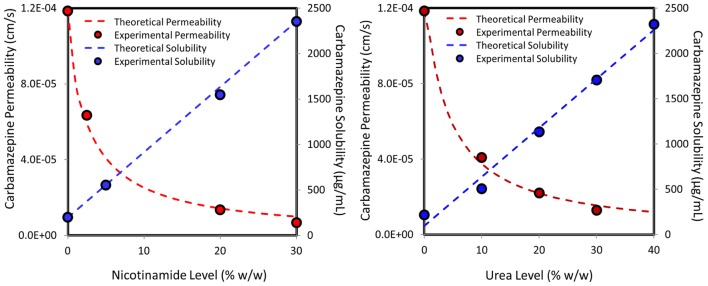
**Experimental (circles) vs. theoretical (lines) solubility (blue) and *in vivo* permeability (red) of carbamazepine as a function of increasing nicotinamide (left panel) or urea (right panel) levels in the single-pass intestinal perfusion model, illustrating the solubility–permeability tradeoff when using hydrotropic drug solubilization**.

To fully capture the simultaneous effects of hydrotropic drug solubilization on the solubility and the permeability, the correlation between the nicotinamide/urea level and the resulted carbamazepine apparent solubility–permeability were modeled. In both cases, the decreased permeability was a mirror-image of the increased solubility of carbamazepine as a function of hydrotrope concentration. This point encouraged us to estimate carbamazepine’s membrane permeability at a given hydrotrope level from the proportional increase in the apparent solubility of the drug. Basically, we plotted the theoretical permeability decrease as a function of hydrotrope concentration, according to the solubility enhancement afforded by this hydrotrope level, according to the following equation:

$$P_{\text{m}}=\frac{P_{\text{m}\left(\text{o}\right)}S_{\text{aq}\left(\text{o}\right)}}{S_{\text{aq}}}$$

where *P*_m_, the apparent intestinal membrane permeability at a given hydrotrope level is equal to the intrinsic membrane permeability of the drug in the absence of hydrotrope (*P*_m(o)_) adjusted to the ratio between the intrinsic solubility, that is, carbamazepine’s solubility in the absence of hydrotrope (*S*_aq(o)_) to the apparent solubility obtained at the same hydrotrope level (*S*_aq_). The resulted theoretical (vs. experimental) solubility–permeability interplay as a function of increasing nicotinamide (left panel) or urea (right panel) concentrations is illustrated in **Figure 4**. The agreement between the calculated and the experimental values is noticeably confirmed; this figure clearly demonstrates the opposing concomitant effects of hydrotropic drug solubilization on the drug’s apparent solubility and intestinal permeability.

## Discussion

Low water solubility is a major concern in todays’ biopharmaceutics. While hydrotropy may assist to increase the apparent solubility of a lipophilic drug, the effect of this solubilization method on the drug’s permeability has not been investigated yet. The focus of this work was to evaluate the solubility–permeability interplay when using hydrotropy in solubility-enabling formulations for lipophilic drugs. It was revealed that, similarly to cyclodextrins, surfactants, and cosolvents, and unlike ASD formulations, hydrotropy is associated with a solubility–permeability tradeoff phenomenon, which means that the solubility gain afforded by the formulation has the “price” of concomitant permeability loss. This finding significantly affects the use of hydrotropic drug solubilization, as it is now clear that the solubility–permeability interplay cannot be ignored throughout the formulation development process; it is not enough to allow the highest solubility possible, rather, the optimal solubility–permeability balance should be aimed in order to maximize the overall oral drug absorption.

In our previous reports we have detected a fundamental mechanistic reason that governs the solubility–permeability interplay nature; while cyclodextrins-, surfactants-, and cosolvents-based solubilization modify the equilibrium aqueous solubility of the drug, supersaturation achieved by ASD formulation is a non-equilibrium/kinetic increase of the drug’s apparent solubility (Miller et al., 2012a; Dahan et al., 2013a). Since the equilibrium aqueous solubility of the drug controls its membrane/aqueous partition coefficient, which in turn controls the drug’s (passive) intestinal permeability, increased equilibrium solubility inherently decreases the drug’s partitioning and the overall permeability, resulting in the solubility–permeability tradeoff obtained with cyclodextrins, surfactants and cosolvents (Miller and Dahan, 2012; Beig et al., 2013b). On the other hand, ASD formulations that allow to achieve and maintain supersaturation which is a non-equilibrium increase of the drugs’ apparent solubility, do not affect neither the drug’s partitioning not its permeability, circumventing the complication of the solubility–permeability tradeoff (Dahan et al., 2016). This analysis highlights that the data presented in this article unequivocally indicate that the mechanism for hydrotropic drug solubilization involves modification of the equilibrium aqueous solubility of the drug. Since the exact mechanism of hydrotropy is not yet fully understood, this finding may contribute to the overall understanding of hydrotropic drug solubilization.

The main structural characteristic of hydrotropes includes hydrocarbon fragment and an ionic moiety. This amphiphilic molecular structure is assumed to play a role in hydrotropic drug solubilization (Li et al., 2015; Patil et al., 2015). The hydrotrope molecules are assumed to self-aggregate by a stacking mechanism, in a manner closely related to surfactants (Bauduin et al., 2005). However, the effect of hydrotropy on the surface tension and other parameters was reported to significantly differ from surfactants, indicating that hydrotrope self-aggregation may not be as central as with micellar solubilization (Balasubramanian et al., 1989; Horváth-Szabó et al., 2001). Changing the solute-water interaction by changing the solvent’s ability to join structure formation through intermolecular hydrogen bonding was also suggested as a potential mechanism for hydrotropy (Coffman and Kildsig, 1996a,b), however, recent enthalpy calculations have doubted the role of this mechanism as well (Shimizu et al., 2013). Another proposed mechanism includes hydrotrope molecules fit around the solute, turning its solvation more susceptible (Hussain et al., 1993; da Silva et al., 1999), although the drug and the hydrotrope may not have a direct mutual attraction, rather, they interact to minimize their contact with water (Sanghvi et al., 2007). Overall, it is likely that the combination of several mechanisms together, rather than one central process, is responsible for the powerful hydrotropic drug solubilization. The biphasic solubility enhancement revealed with urea (**Figure 1**, left) also supports the idea of a combination of different mechanisms; it is likely that at lower urea concentration one mechanism is active, and at a critical hydrotrope level (10% w/w in our case) an additional mechanism kicks in and enhances the solubilization capacity, resulting in the biphasic trend. Hydrotropic solubilization using nicotinamide showed no such biphasic effect (**Figure 1**, right panel), suggesting that the different dominant mechanisms of hydrotropy may differ from one hydrotrope to another; similar slopes were obtained for the hydrotropic solubilization by urea above 10% at 37°C and by nicotinamide at all conditions, suggesting similar predominant solubilization mechanisms for these two hydrotropes under certain conditions, but not necessarily under other conditions (e.g., at room temperature or below 10% for urea). This point is further highlighted by the very different reaction to temperature of the two studied hydrotropes: while the ability of urea to increase carbamazepine solubility had a strong temperature-dependency, it was revealed that nicotinamide can produce its full 37°C solubilization capacity already at room temperature. Revealing that hydrotropy influences the drugs’ equilibrium solubility may also aid to elucidate the underlying mechanism(s) of hydrotropy.

An excellent agreement was achieved between the experimental permeability data and the predicted values, both *in vitro* and *in vivo* (**Figure 4**), although a relatively simplified prediction approach was taken. We have shown before that in cases where the unstirred water layer (UWL) adjacent to the intestinal wall plays a significant role as an absorption barrier for lipophilic drugs, this simplified approach may not be sufficient to allow this level of prediction, and a more complicated quasi-equilibrium transport analysis may be needed (Dahan et al., 2010b; Miller et al., 2011, 2012b). For carbamazepine, we have shown previously that the UWL does not act as a significant permeation barrier (Beig et al., 2012), and hence the simplified approach presented in the paper, that involves a proportional opposite correlation between the solubility increase and the permeability decrease at a given hydrotrope concentration, allowed excellent prediction of the overall solubility–permeability interplay (**Figure 4**).

## Conclusion

Hydrotrope-based solubility-enabling formulation can result in significant water solubility increase, but at the same time may cause intestinal permeation decrease. Hence, when using hydrotropic drug solubilization it is prudent to not focus solely on the solubility, but to account for the permeability as well. Achieving optimal solubility–permeability balance may promote the overall goal of the formulation to maximize drug exposure following oral administration.

## Author Contributions

Conceived and designed the experiments: AB, DL, JM, RA, and AD. Performed the experiments: AB and AD. Analyzed the data: AB, DL, JM, RA, and AD. Wrote the paper: AB and AD.

## Conflict of Interest Statement

The authors declare that the research was conducted in the absence of any commercial or financial relationships that could be construed as a potential conflict of interest.
